# Creative, Socially-Minded Communication Project on Neurodivergences Increases Self-Reported Content Knowledge, Collaboration Skills, and Personal Allyship

**DOI:** 10.59390/001c.162900

**Published:** 2026-06-04

**Authors:** G. Daniel Holmes, Deborah E. Bauer, Sharon M. H. Gobes

**Affiliations:** 1 Neuroscience Wellesley College

**Keywords:** neurodiversity, science communication, outreach, student-centered pedagogy, culturally relevant pedagogy, authentic learning project

## Abstract

Science communication, social consciousness, and collaboration are all essential for ethical, public-minded science. This zine-based communication project presents an opportunity for students to engage in authentic communication whilst developing their social, neurological, and justice-oriented understandings of neurodivergences. The project employed hands-on, team-based learning; generative writing-to-learn and writing-to-teach methodologies, augmented by the inclusion of artistic design; and authentic purpose and audience engagement. The success of the project was evaluated through self-assessment surveys, which students completed prior to and following the conclusion of the project. Combined quantitative and qualitative analysis of these surveys demonstrates that students made significant gains in knowledge of both the neurological and social aspects of neurodivergences across multiple domains. Students reported increases in depth of knowledge, as well as development of personal allyship, communication skills, and collaboration ability. The Zine Project may be implemented in undergraduate courses to support students in gaining these critical skills.

Educational theorists have long held that the scientific community has an ethical obligation towards accurate, effective, and community-conscious engagement and communication—a necessity that has become more readily apparent in the aftermath of the COVID-19 pandemic (Dewey, 1927; Leshner, 2003; Matta, 2020). Community engagement is a core goal for many post-secondary institutions (Morin et al., 2016); researchers and funding bodies alike endorse participation in science communication as critical for the success of scientific enterprises, from the classroom to broadscale initiatives (e.g. NSF’s ‘Broader Impacts’ merit criterion). Furthermore, there is an increasing emphasis on interactions and communication with non-experts, particularly as that work pertains to making science more ethical, social-justice-oriented, and responsive to community needs and values (Rose et al., 2020).

Furthermore, management and communication skills are valued across life sciences career paths, as well as by graduate students and advisors (Shah & Juavinett, 2022), and written communication in the sciences is considered a core competency for undergraduate neuroscience and neuroscience careers (AACU, 2007; Akil et al., 2016; Woodin et al., 2010). Specifically, critical and integrative thinking, written and visual communication skills, and an appreciation of how neuroscience can contribute to solving societal problems are now held as necessary components of an effective, inclusive, forward-facing neuroscience pedagogy (Ramirez, 2020).

In order to develop these skills, students benefit from hands-on, authentic learning projects. Pedagogical structures that involve the creation of a novel idea or synthesis are considered one of the most effective tools for developing students’ long-term skill and content knowledge (Anderson & Krathwohl, 2001; Bloom, 1956; Krathwohl, 2002). Furthermore, artistic, creative approaches to teaching scientific concepts can deepen learning and engage different dimensions of thinking; incorporating artistic methodologies can be used to promote “transformative creativity,” which refers to the ability to develop new methodologies and approaches to scientific questions (Hake, 1998; Lehmann & Gaskins, 2019).

Despite their strong theoretical grounding, writing-to-learn and writing-to-teach paradigms are only recently gaining traction in undergraduate neuroscience curricula. These approaches have shown significant efficacy in developing students’ deeper conceptual understanding of scientific concepts and socio-scientific issues (Balgopal et al., 2018; Reynolds et al., 2012; Vázquez et al., 2012).

Another critical dimension of developing projects that target socio-scientific understanding is authenticity to community and student body. Authentic science communication projects are positively associated with student confidence (Cirino et al., 2017). Additionally, such projects—those that have genuine purposes related to the goals and values of the students—are uniquely positioned to integrate culturally sustaining pedagogies, develop students’ nature of science understandings and facilitate epistemic cognition.

“Culturally sustaining” or “culturally relevant pedagogy” is a framework for teaching that centers not only academic content or skills but also cultural and sociopolitical competencies. It intentionally works to advance equity, build relationships, and cultivate an anti-oppressive practice, valuing students’ lived experiences and integrating the cultural context of the educational system into teaching in a way that aims towards liberation (Berthoff, 1990; Ladson-Billings, 1995, 2014). This framework is critical in the context of socio-scientific issue education, to develop students’ understandings of the nature of science in its political, historical, cultural, and social contexts, and to actively dismantle systems of oppression (Berthoff, 1990; Lederman et al., 2013; Leung, 2022; Zeidler & Keefer, 2003).

Zines are short, hand-designed magazines combining text and graphical elements, historically with a social or political purpose. While zine-making has long been a staple of the humanities, its application in STEM education is not widespread (Buchanan, 2012; Congdon & Blandy, 2003; Thomas, 2018; Williamson, 1994). Zines that convey curricular material to students were adopted in high school chemistry classrooms, resulting in increased content-based performance and enhanced enjoyment in learning (Cook & van Hest, 2024). When students create science zines, it deepens their knowledge of a topic and requires them to translate technical jargon into public-facing narratives, which enhances conceptual understanding and science communication (“participatory science literacy”, Yang, 2010). Despite their potential, zines remain underutilized in interdisciplinary or transdisciplinary learning, including when teaching STEM-related social justice and advocacy (see Brown et al., 2021 for one example of this pedagogy in STEAM in an out-of-school setting).

The Zine-based curriculum presented here engaged students in an authentic, culturally relevant science communication scenario, during which they developed skills in collaboration, socio-scientific integration, and creative communication. The motivation to develop this zine-based curriculum was that the core curriculum for our Neuroscience major provided limited content knowledge on neurodivergences (one lecture on “Autism” in our 200-level course) or the neurodiversity movement, which views previously-pathologized differences in brain function and behavior as normal variation in the human population (Fung & Doyle, 2021). Throughout the project, students worked in small groups to craft zines on the topic of a neurodiverse condition for distribution to a local middle school. Students explored the neurological and societal contexts of various neurodivergences, while learning about the ways in which the science of neurodiversity has been shaped by cultural and societal expectations and narratives. Together, these elements allowed students to develop their understanding of neuroscience and communication through a personally meaningful medium, augmenting their knowledge and skill gains.

## MATERIALS AND METHODS

Participants were students (n = 33) at Wellesley College, a small liberal arts college in the Northeastern United States serving women and other minoritized genders. Students were senior neuroscience majors enrolled in the Capstone Seminar in Neuroscience, which is mandatory for majors at Wellesley College; students were distributed across three sections of the course (one section of 9 and two sections of 12), each taught by a different professor with their own interpretation of the lesson plans. All students had completed two core courses in the neuroscience department, both including laboratory work, in addition to an introductory cellular and molecular biology laboratory course. Two students of the original 33 opted out of giving consent for the use of their survey data, and 3 students failed to complete the pre-survey. The Brandeis University IRB (which oversees all human subject research at Wellesley College) deemed this human subjects protocol exempt in accordance with 45 CFR 46.104(d)(1), under protocol #25037R-E to S.M.H. Gobes).

### Classwork & Project

The Zine Project was introduced to students as a creative expression of a research project on neurodivergences with an advocacy component. Students were told the purpose of the zines: to serve as educational tools for middle-school aged children, offering insight into the neurological aspects of various conditions while highlighting the strengths and unique perspectives of neurodivergent individuals. Prior to being introduced to the Zine Project, students completed a self-report questionnaire consisting of a combination of free response and rating scale questions intended to assess students’ attitudes, skills, learning, and knowledge development (Supplementary Material 1A). Students received credit for completing the questionnaire but were told the content of their answers would not affect their grade in the course as the survey was anonymous.

The concept of neurodiversity was introduced through the assignment’s first learning goal: to “demonstrate general knowledge of neurodiversity. Students should be able to define and explain the concept of neurodiversity, understanding that it encompasses the natural variation in neurological conditions, cognitive abilities, and behavioral traits within the human population”. The concept was further defined through an assigned reading of the online text: ‘What is Neurodiversity’ by the Child Mind Institute (https://childmind.org/article/what-is-neurodiversity/). The assignment was scaffolded with four workshops, each 75 minutes, spread throughout the semester:

1. a workshop with the science librarian on effective research methods and reliable resources.
2. introduction to zines (what zines are) and to the audience (middle school children), and a workshop on successful group work, including creating a group contract. During this workshop, the teams were formed based on interest in a specific topic (choice of: Attention-Deficit /Hyperactivity Disorder, anxiety, Autism Spectrum Disorder, dyscalculia, dyslexia, Obsessive Compulsive Disorder, or a different neurodivergence suggested by the students) and the skill they bring to the table (e.g. creative design, background research etc).

In between workshops 2 & 3, students were tasked with doing their background research on the topic and creating an outline of the information that they intended to share with their audience (approximately 5 hours of out-of-class work) in preparation for the next workshop:

1. discussion of the zine format (i.e. what is effective & what is not) in small groups based on examples of zines on different topics.

Between workshop 3 & 4, students created the first version of their zine (approximately 10 hours of out-of-class work) and brought it to class for the fourth and final session:

1. peer review, in which students gave each other feedback in a Jigsaw format using peer feedback forms.

At the conclusion of the course, and after students handed in their final Zine, they completed a second anonymous questionnaire (Supplementary material 1B). Outside of the workshops, no class time was allotted to the Zine Project or to covering content related to neurodivergences and neurodiversity. No instructor feedback was provided except in-class while walking around the tables. Other assignments in the course centered around writing a mock NIH F31 grant proposal, conducting Journal Clubs on articles written by visiting guest speakers, and exploring career opportunities.

The Zine assignment instructions for students, lesson plans, and all other teaching materials are available on request at this Google Drive link: https://drive.google.com/drive/folders/1ehnuidaa5iBR0MwZJRDZoRctyOI8DxiI?usp=sharing.

### Data analysis

#### Matched pre- and post- questions

Paired t-tests were conducted on matched pre- and post-project questionnaire ratings. An alpha level of 0.05 was used.

#### Other quantitative measures

All possible combinations of quantitative measures were analyzed for correlation using the Bayesian Pearson Correlation function in SPSS; p-values were corrected for multiple comparisons using the Benjamini-Hochberg correction, also conducted in SPSS. A post-correction alpha level of 0.05 was used.

#### Qualitative analysis

Qualitative responses were collated between pre- and post-surveys based on anonymized participant codes. They were then processed via a combined grounded theory and framework-based thematic analysis approach.

First, survey responses were tagged in Taguette using a combination of predetermined thematic categories based on the learning goals and content objectives outlined in the project description and emergent categories based on patterns observed during tagging (tags available in Supplementary Material 2). Analytical memos describing emerging themes were written throughout grounded-theory data analysis (Birks, 2008). Following the initial coding process, all content with shared tags was collated and reanalyzed for patterns within the categories, allowing for characterizations of internal patterns. These patterns were then used to develop final theme categories and produce a proposed narrative mapping of student experience of, and learning via, the Zine Project.

## RESULTS

### Students reported gains in knowledge of the social and neurological aspects of neurodiversity

**Figure 1.**
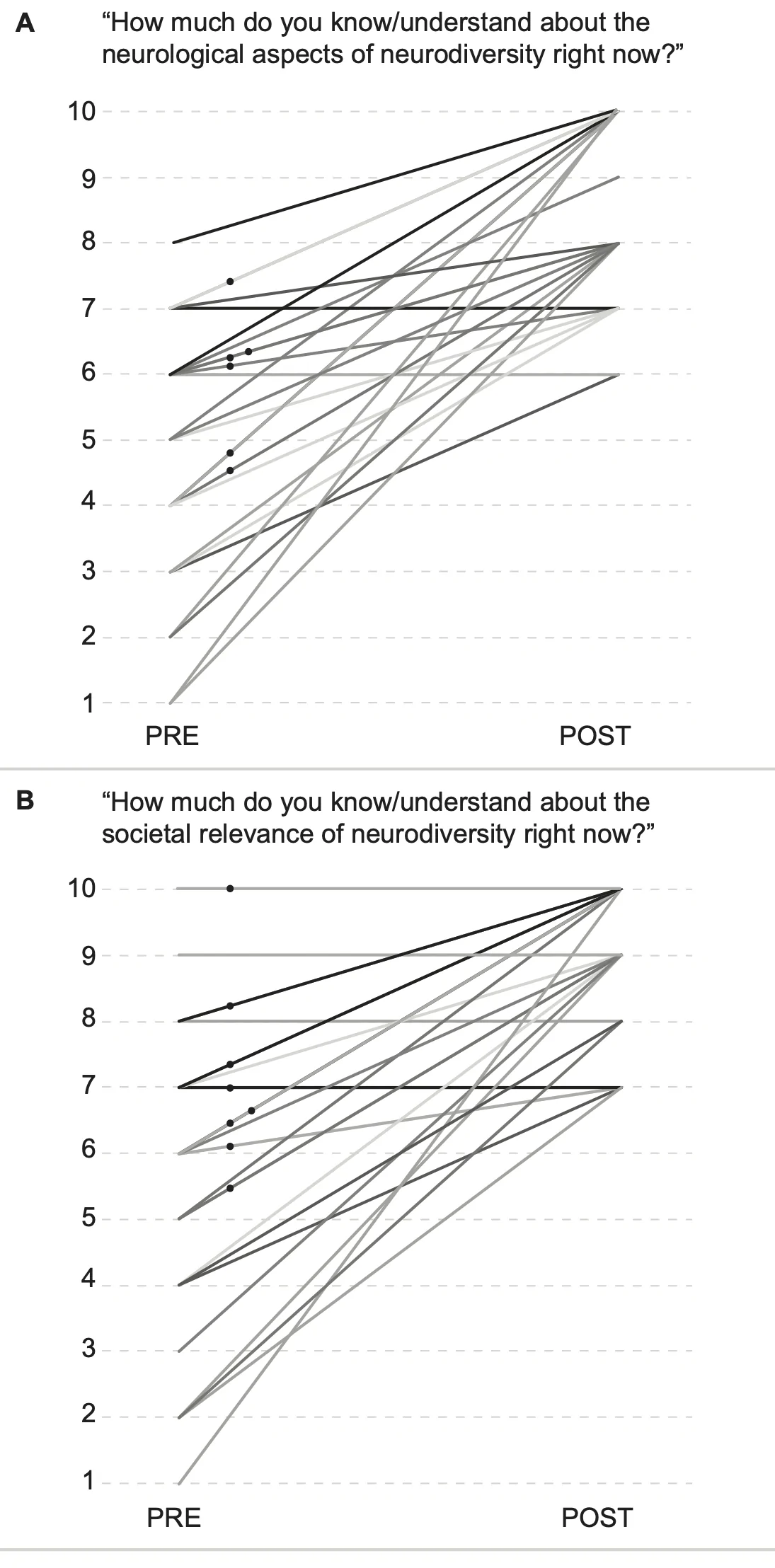
Students reported gains in content knowledge of both neurological and social aspects of neurodiversity. Responses aspects of neurodiversity right now?” (A) and “How much do you know/understand about the societal relevance of neurodiversity right now?” (B). Each line represents pre/post answer values from a single student and overlapping lines from multiple students with the same values are represented with a circle for each additional student. Both comparisons were significant at a threshold of < 0.001 per paired t-test.

Students completed surveys prior to and following completion of the Zine Project, about 3 months apart. Based on their appraisals of their own knowledge at each timepoint, the project led to significant increases in knowledge of both the neurological (Fig. 1A) and societal (Fig. 1B) aspects of neurodiversity. This demonstrates that students increased their confidence in their knowledge of the topic, an interpretation that is further supported by students’ own reflections on the post-project survey: when asked to rate their improvement in understanding on a 1 to 10 Likert scale, students reported a mean of 7.5 (SD = 2.25). These gains in understanding were primarily in the domains of neuroanatomical correlates of neurodiversity, social movements, medical context, and presentation, as determined by thematic analyses of open-ended survey responses (Table 1). Though students reported learning about a multitude of themes, many noted in their surveys that they primarily explored these broader notions solely through the lens of their specific zine’s topic, or that they had the opportunity to learn a lot about certain particular ideas but not neurodivergence overall. A quantitative tally of instances where students described an increase in depth of knowledge or knowledge solely on a specific sub-topic vs. instances in which students cited an increase in knowledge of neurodiversity more generally showed that a greater proportion of students noticed improvements in depth (72.4%), rather than breadth (37.9%), of knowledge.

**Table 1.** Students gained content knowledge across scientific and social domains. Open-ended survey responses coded as “content knowledge description” were analyzed for major themes, which are listed in the leftmost column. Incidence was counted as the number of individual survey responses (n = 30) that described a given theme. Representative quotes are shown in the rightmost column; some quotes may pertain to multiple themes and were tallied as such for the purposes of “incidence”.

**Theme**	**Incidence**	**Representative Quotations**
Societal Dimensions	12	I learned more about…societal aspects like representation of neurodiversity, common myths, and stigmas. It allowed me to understand neurodiversity on a much deeper level, knowing the symptoms and especially its impact on society. I think that I know more about some of the myths and stereotypes that surround neurodiversity that I was not even aware existed prior to this assignment. I learned a lot about […] its effect on people within society through factors such as harmful stereotypes, lack of research, symptoms, etc.
Neuroanatomy	10	It was helpful for understanding brain regions that may be responsible for certain traits associated with neurodiversity. I was able to understand the neural pathways and areas of the brain that are implicated.
Medical Context	9	I did not have an updated perspective on the diagnosis of this type of neurodivergence until this assignment. I achieved solid baseline understandings of a variety of neurodiverse conditions - ranging from history of diagnosis to treatment options to stereotypes and attitudes.
Presentation & Behavior	5	I learned a lot about different presentations of neurodiversity. I didn't realize how big masking was until this project, but now I understand that a lot of people may never be diagnosed simply because they got so used to trying to fit in to a neurotypical society.
Cellular & Molecular Mechanisms	3	I feel like I have learned a decent amount about the biological, chemical, and neural components of diversity. I learned more about the specific theories behind neurobiological explanations for these disorders.

### Advocacy and tangible change were foregrounded in personal learning, whereas appreciation and breaking down stigma were priorities for communication

**Figure 2.**
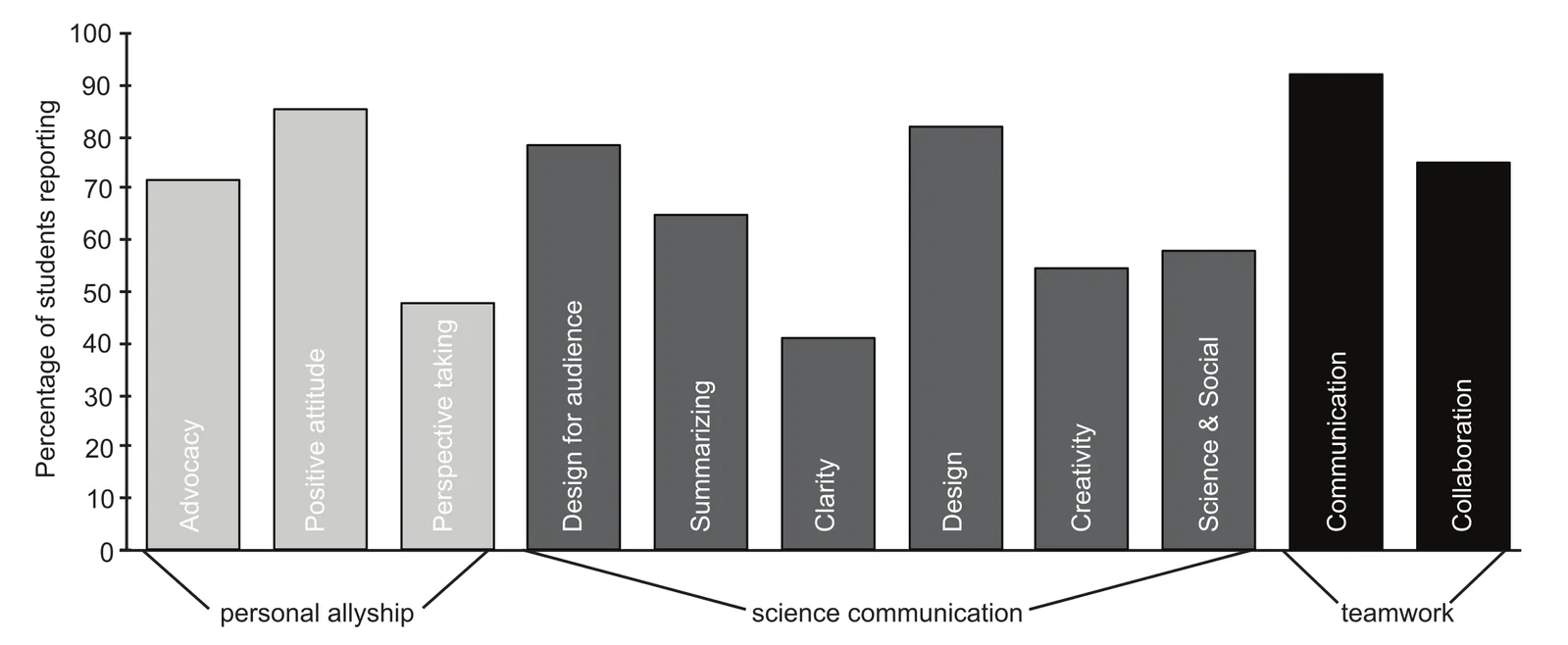
Student responses revealed gains in personal allyship, science communication skills, and teamwork. Open responses with matched pre- and post- data (n = 28) were analyzed, coded, and categorized, yielding 11 themes referring to positive developments over the course of the project. The percentages of students demonstrating or reporting improvement or positive attitudes encompassed by each of these themes are shown above. Within the “personal allyship” domain, advocacy refers to an intention to advocate for neurodivergent individuals in future work/contexts; positive attitude refers to positive attitudes towards neurodivergence or neurodiversity; and perspective-taking refers to expressed empathy for various stakeholders (neurodivergent individuals, clinicians, families, fellow students, middle-schoolers, etc.). Within the “science communication” domain, design for audience refers to considering the audience when making choices around content and presentation; summarizing refers to gathering information from multiple sources and creating a clear and cohesive abridged description; clarity refers to the skill of crafting accessible, concise, and appropriately detailed work; design refers to the skill of evaluation and crafting visual, spatial, and tactile elements of a work; creativity refers to creative problem-solving and unexpected, novel choices; and science & social refers to the ability to articulate the scientific and social dimensions of a subject in tandem. Within the “teamwork” domain, communication refers to sharing information with and receiving feedback from peers, professors, and others within the college community; collaboration refers to the other skills necessary for effective teamwork (e.g. managing team dynamics, dividing work, reaching consensus, etc.).

Multiple students described a desire to pursue further projects where they would educate people about and advocate for neurodivergent communities. In some cases, this theme was developed through a focus on advocating for increased accommodations and access to support. Several students reported a shifting mindset from neurodivergence as a negative to a positive attribute, and others described developing understandings of their own neurodivergence and that of those around them. Over 70% of student responses described an intention towards future advocacy or education work (Fig. 2).

In describing their goals for communicating with youth through the Zine Project, students noted a desire to break down stigma, counter stereotypes, or otherwise increase acceptance and appreciation for neurodiversity.

### The Zine Project developed student research, communication, and collaboration skills

In addition to the personal development students described, many cited improvement in key dimensions of science communication, including designing communications for a specific audience (79%), isolating and summarizing key information (65%), communicating clearly (41%), using graphical design elements (82%), creatively conveying information (55%), and integrating scientific and societal information (58%). Many students also described practicing and improving skills in collaboration (76%) (Fig. 2).

### Post-project knowledge and skill gains were not predicated on pre-project knowledge or interest level

There were no significant correlations between pre-project variables, including prior interest in neurodiversity, previous Zine creation experience, or outreach, and post-project self-evaluation of project success and benefit. Similarly, prior knowledge of neurodiversity topics did not correlate positively or negatively with any of the quantitative outcome variables on the post-project survey. That said, responses to post-project survey questions asking students to rate their contributions to and experience with the collaborative process were significantly and positively correlated with student ratings of improvement in transferable skills (p = 0.002, R = 0.542.), success in achieving project objectives around creativity (p < 0.001, R = 0.630), and improvements in understanding (p = 0.002, R = 0.517).

## DISCUSSION

The Zine Project effectively increased students’ perceived understandings of the neurological and social aspects of specific neurodivergences while developing their self-reported abilities to communicate science, collaborate with teammates, and view neurodiversity from a social justice lens.

Students’ content learning spanned multiple domains of knowledge. However, despite this variety, students primarily reported increased depth of knowledge, rather than increased breadth. Given research demonstrating that increased depth of learning, rather than simply greater amounts of content covered, leads to gains in future preparedness, this self-reported observation bodes well for students’ careers (Ramirez, 2020; Schwartz et al., 2008).

Student responses also demonstrate the project’s ability to drive students’ integration of social and neurological domains. Critically, engaging students in epistemic cognition—linking their learning about science deeply and inherently to an understanding of the social nature of science, while encouraging metacognitive reflection—is one of the key ways that science teaching can be integrated with culturally relevant pedagogy. To truly develop nature of science understanding such that it can then be applied to socio-scientific contexts, students must develop knowledge of both historical and ongoing scientific inquiry and the epistemic uncertainties involved with and resolved by each (Leung, 2022).

Furthermore, perspective-taking and integration of social and scientific contexts are both critical to ethical science communication and engagement. This integration aids students in merging emotional and social dimensions with content, which augments student learning and ability to implement school-based learning during real-world decision-making and action (Immordino-Yang & Damasio, 2007).

Pre- and post- measures of interest, learning, and prior knowledge/skills were not correlated; this encouraging result indicates that the project is effective in increasing student understanding and skills regardless of prior interest, knowledge, or expectation. This demonstrates that the Zine Project can lead to meaningful gains regardless of initial student buy-in.

That said, there were significant correlations between measures of collaboration and learning. As such, future implementations must prioritize effective teamwork—many students cited the creation of teamwork contracts and specific expectations as supportive in this endeavor. It is also important to note that class norms may be helpful, both in supporting collaboration and avoiding concerns around class sensitivity. The nature of the project as a culturally responsive, authentic environment requires engagement with difficult topics that may open the door for microaggressions, as was mentioned in student responses for this implementation; clear class expectations and discussion around respect may help to avoid this concern in the future.

Furthermore, additional feedback from both the instructor and peers will help students feel more supported. In subsequent renditions of this course, using the same zine assignment on a novel topic (not neurodiversity), we have made two adjustments in providing feedback. First, the instructor now provides feedback on the ‘outline’ generated by students after they have done their initial research. Second, peer reviewers leave asynchronous feedback on the *first* draft of the zine, and on the *second* draft of the zine via the in-class Jigsaw activity, followed by a discussion led by the instructor. An additional change that was incorporated in subsequent renditions was using zines from students who had previously taken the course as examples to critique (zines can be provided upon request at this Google Drive link: https://drive.google.com/drive/folders/1e__0CM5L6A07Lbn7f2QiEYT2D-xkJh4N). Both of these changes seemed to improve students’ perception of receiving sufficient support to successfully complete the assignment.

Future implementations should also consider choosing additional topics based on the interests and needs of the specific student population. The core of culturally responsive pedagogy is creating authentic learning opportunities that are integrated with students’ real concerns and lives; as such, exploring the neuroscience of topics beyond neurodiversity such as gender, racial justice, or economic inequality based on which are most important to one’s target population would align well with the spirit and purpose of the assignment.

### Address correspondence to:

Sharon M. H. Gobes, Professor of Neuroscience, Neuroscience Department, Wellesley College, 106 Central Street, Wellesley, MA 02481. Email: sgobes@wellesley.edu

## Supplementary Material

Supplementary material 1A

Supplementary material 2

Supplementary material 1B

## References

[ref-591239] AACU (2007). College learning for the new global century.

[ref-591237] Akil H., Balice-Gordon R., Cardozo D. L., Koroshetz W., Posey Norris S. M., Sherer T., Sherman S. M., Thiels E. (2016). Neuroscience Training for the 21st Century. Neuron.

[ref-591238] Anderson L. W., Krathwohl D. R. (2001). A Taxonomy for Learning, Teaching, and Assessing: A Revision of Bloom's Taxonomy of Educational Objectives.

[ref-591240] Balgopal M. M., Casper A. M. A., Wallace A. M., Laybourn P. J., Brisch E. (2018). Writing Matters: Writing-to-Learn Activities Increase Undergraduate Performance in Cell Biology. BioScience.

[ref-591241] Berthoff A. E. (1990). Paulo Freire's Liberation Pedagogy. Language Arts.

[ref-591243] Bloom B. S. (1956). Taxonomy of Educational Objectives.

[ref-591244] Brown A., Hurley M., Perry S., Roche J. (2021). Zines as Reflective Evaluation Within Interdisciplinary Learning Programmes. Front. Educ..

[ref-591245] Buchanan R. (2012). Zines in the Classroom: Reading Culture. Engl. J..

[ref-591246] Cirino L. A., Emberts Z., Joseph P. N., Allen P. E., Lopatto D., Miller C. W. (2017). Broadening the voice of science: Promoting scientific communication in the undergraduate classroom. Ecology and Evolution.

[ref-591247] Congdon K. G., Blandy D. (2003). Zinesters in the Classroom: Using Zines to Teach about Postmodernism and the Communication of Ideas. Art Educ.

[ref-591248] Cook A. B., van Hest J. C. M. (2024). Comic Zines as Tools for Chemistry Education and Engaging Students. J Chem Educ.

[ref-591249] Dewey J. (1927). The Public and Its Problems.

[ref-591250] Fung K. L., Doyle N., Fung L. K. (2021). Neurodiversity: From Phenomenology to Neurobiology and Enhancing Technologies.

[ref-591251] Hake R. R. (1998). Interactive-engagement versus traditional methods: A six-thousand-student survey of mechanics test data for introductory physics courses. American Journal of Physics.

[ref-591252] Immordino-Yang M. H., Damasio A. (2007). We Feel, Therefore We Learn: The Relevance of Affective and Social Neuroscience to Education. Mind, Brain, and Education.

[ref-591253] Krathwohl D. R. (2002). A revision of Bloom's taxonomy: An overview. Theory into practice.

[ref-591255] Ladson-Billings G. (1995). Toward a Theory of Culturally Relevant Pedagogy. American Educational Research Journal.

[ref-591254] Ladson-Billings G. (2014). Culturally Relevant Pedagogy 2.0: A.k.a. the Remix. Harvard Educational Review.

[ref-591256] Lederman N. G., Lederman J. S., Antink A. (2013). Nature of Science and Scientific Inquiry as Contexts for the Learning of Science and Achievement of Scientific Literacy. Science and Technology.

[ref-591257] Lehmann J., Gaskins B. (2019). Learning scientific creativity from the arts. Palgrave Communications.

[ref-591258] Leshner A. I. (2003). Public Engagement with Science. Science.

[ref-591259] Leung J. S. C. (2022). A Practice-Based Approach to Learning Nature of Science through Socioscientific Issues. Research in Science Education.

[ref-591260] Matta G. (2020). Science communication as a preventative tool in the COVID19 pandemic. Humanities and Social Sciences Communications.

[ref-591261] Morin S. M., Jaeger A. J., O'Meara K. (2016). The State of Community Engagement in Graduate Education: Reflecting on 10 Years of Progress. Journal of Higher Education Outreach and Engagement.

[ref-591262] Ramirez J. J. (2020). Undergraduate neuroscience education: Meeting the challenges of the 21st century. Neuroscience Letters.

[ref-591263] Reynolds J. A., Thaiss C., Katkin W., Thompson R. J. (2012). Writing-to-Learn in Undergraduate Science Education: A Community-Based, Conceptually Driven Approach. CBE—Life Sciences Education.

[ref-591264] Rose K. M., Markowitz E. M., Brossard D. (2020). Scientists' incentives and attitudes toward public communication. Proceedings of the National Academy of Sciences.

[ref-591265] Schwartz M. S., Sadler P. M., Sonnert G., Tai R. H. (2008). Depth vs. Breadth: How Content Coverage in High School Science Courses Relates to Later Success in College Science Coursework. Science Education.

[ref-591266] Shah S., Juavinett A. L. (2022). The Mismatch Between Neuroscience Graduate Training and Professional Skill Sets. Journal of Undergraduate Neuroscience Education.

[ref-591267] Thomas S. (2018). Zines for Teaching: A Survey of Pedagogy and Implications for Academic Librarians. Portal Libr. Acad..

[ref-591268] Vázquez A. V., McLoughlin K., Sabbagh M., Runkle A. C., Simon J., Coppola B. P., Pazicni S. (2012). Writing-To-Teach: A New Pedagogical Approach To Elicit Explanative Writing from Undergraduate Chemistry Students. Journal of Chemical Education.

[ref-591269] Williamson J. (1994). Engaging Resistant Writers through Zines in the Classroom.

[ref-599941] Woodin T., Carter V. C., Fletcher L. (2010). Vision and change in biology undergraduate education, a call for action--initial responses. CBE Life Sciences Education.

[ref-591271] Yang A. (2010). Engaging Participatory Literacy through Science Zines. Am. Biol. Teach..

[ref-591272] Zeidler D. L., Keefer M., Zeidler D. L. (2003). The Role of Moral Reasoning on Socioscientific Issues and Discourse in Science Education.

