# Supplementary material for: Creative, Socially-Minded Communication Project on Neurodivergences Increases Self-Reported Content Knowledge, Collaboration Skills, and Personal Allyship
Source: J Undergrad Neurosci Educ. 2026 Jun 4;24(2):71–9. doi: 10.59390/001c.162900 (PMC13625903; doi:10.59390/001c.162900)
Supplement: Supplementary file 1 [file junejournal_2026_24_2_162900_347744.pdf]

# pre-survey Zines (Neurodiversity)

* Indicates required question

1. Anonymous identifier to connect pre & post data, created from the initials from your best friend's name (XX) followed by a 6-digit number that is your best friend's birthday (MMDDYY). For example RS120602: *

2. Your section of NEUR300: *

   Mark only one oval.

   - Prof. A
   - Prof. B
   - Prof. C
   - Prof. D
   - Other:

3. Briefly explain your understanding of neurodiversity and its significance before starting this assignment. *

4. How much do you know/understand about the neurological aspects of neurodiversity right now? *

   Mark only one oval.

	1	2	3	4	5	6	7	8	9	10	
noth											a lot

5. How much do you know/understand about the societal relevance of neurodiversity right now? *

   Mark only one oval.

	1	2	3	4	5	6	7	8	9	10	
noth											a lot

6. What do you hope to gain from participating in the Zine Outreach Project on neurodiversity? *

7. Before starting the Zine Outreach Project, rate how much you expect to learn about neurodiversity concepts through this assignment. *

   Mark only one oval.

   - none
   - a little
   - a moderate amount
   - a substantial amount

8. How interested are you in the subject of neurodiversity? *

Mark only one oval.

- not at all
- a little
- moderately interested
- substantially interested

9. Have you ever created a Zine before? *

Mark only one oval.

- No
- Yes

10. How interested are you in the process of creating a Zine? *

Mark only one oval.

- not at all
- a little
- moderately interested
- substantially interested

11. How interested are you in participating in neuroscience outreach? *

Mark only one oval.

- not at all
- a little
- moderately interested
- substantially interested

12. How motivated are you to collaborate with your team and contribute to the Zine Outreach Project? *

Mark only one oval.

- not at all
- a little
- moderately interested
- substantially interested

Thank you for completing the pre-survey on your expectations with the Zine Outreach Project. Please go to Sakai to submit your anonymous identifier into the quiz. This will allow us to record your credit for participation anonymously.

This content is neither created nor endorsed by Google.

Google Forms
