# Supplementary material for: Creative, Socially-Minded Communication Project on Neurodivergences Increases Self-Reported Content Knowledge, Collaboration Skills, and Personal Allyship
Source: J Undergrad Neurosci Educ. 2026 Jun 4;24(2):71–9. doi: 10.59390/001c.162900 (PMC13625903; doi:10.59390/001c.162900)
Supplement: Supplementary file 2 [file junejournal_2026_24_2_162900_347746.pdf]

# post-survey Zines (Neurodiversity)

* Indicates required question

1. Anonymous identifier to connect pre & post data, created from the initials from your best friend's name (XX) followed by a 6-digit number that is your best friend's birthday (MMDDYY). For example RS060680: *

2. Your section of NEUR300: *

   Mark only one oval.

   - Prof. A
   - Prof. B
   - Prof. C
   - Prof. D
   - Other: ____________

3. What did you learn about neurodiversity and its significance through completing the Zine Outreach assignment? *

4. How much do you know/understand about the neurological aspects of neurodiversity right now? *

   Mark only one oval.

	1	2	3	4	5	6	7	8	9	10	
noth	◯	◯	◯	◯	◯	◯	◯	◯	◯	◯	a lot

5. How much do you know/understand about the societal relevance of neurodiversity right now? *

   Mark only one oval.

	1	2	3	4	5	6	7	8	9	10	
noth	◯	◯	◯	◯	◯	◯	◯	◯	◯	◯	a lot

6. Reflect on your experience with the Zine Outreach Project. How did it contribute to your understanding of neurodiversity? *

7. How much did working with your group on the Zine Outreach Project increase your knowledge/understanding about the neurological aspects of neurodiversity? *

   Mark only one oval.

   - none
   - a little
   - a moderate amount
   - a substantial amount

8. How much did working with your group on the Zine Outreach Project increase your knowledge/understanding about the societal relevance of neurodiversity? *

Mark only one oval.

- none
- a little
- a moderate amount
- a substantial amount

9. Provide an example of how you used your previous knowledge of neuroscience into the zine project. *

10. Provide an example of how you used creative expression to communicate scientific concepts in your Zine. *

11. How do you think your Zine will impact an audience consisting of first-year college students? *

12. Rate your ability to apply skills and knowledge learned in NEUR300 or in the neuroscience major to create a zine for first-year college students. *

Mark only one oval.

	1	2	3	4	5	6	7	8	9	10	
low											exceptional ability

13. How relatable is your zine for first-year college students? *

Mark only one oval.

	1	2	3	4	5	6	7	8	9	10	
not r											exceptionally relatable

14. How informative is your zine for first-year college students? *

Mark only one oval.

	1	2	3	4	5	6	7	8	9	10	
not i											exceptionally informative

15. Did your perspective on neurodiversity change or evolve as a result of this assignment? If yes, please explain. *

16. How much has your perspective on neurodiversity evolved as a result of this assignment? *

Mark only one oval.

- none
- a little
- a moderate amount
- a substantial amount

17. Compare your current understanding of neurodiversity with your initial understanding before starting the assignment. In what ways have you seen growth or changes in your knowledge? *

18. Rate the extent of improvement in your understanding. *

Mark only one oval.

	1	2	3	4	5	6	7	8	9	10	
no ir											significant improvement

19. How did your team collaborate to create an inclusive and informative zine? What aspects of the project worked well for promoting teamwork and inclusivity? What improvements will you make for future team projects? *

20. On a scale of 1 to 10, how effectively did your team collaborate to create an inclusive and informative zine? *

Mark only one oval.

	1	2	3	4	5	6	7	8	9	10	
not (											extremely effective

21. On a scale of 1 to 10, how well did you contribute to and benefit from this collaborative process? *

Mark only one oval.

1	2	3	4	5	6	7	8	9	10

22. Reflecting on the Zine Outreach Project, how do you think it has contributed to your overall ability to communicate complex concepts creatively? *

23. How valuable do you consider the Zine Outreach Project in contributing to your overall ability to communicate complex concepts creatively? *

Mark only one oval.

	1	2	3	4	5	6	7	8	9	10	
not '											extremely valuable

24. What are the skills you learned that may transfer to situations beyond creating Zines on neurodiversity? *

25. How valuable do you consider the Zine Outreach Project in contributing to your improvement in transferable skills? *

Mark only one oval.

	1	2	3	4	5	6	7	8	9	10	
not '	◯	◯	◯	◯	◯	◯	◯	◯	◯	◯	extremely valuable

26. If you would like to elaborate on or explain any of your responses, feel free to do so in the space below.

Thank you for completing the post-survey on your learning experience with the Zine Outreach Project. Please go to Sakai to submit your anonymous identifier into the quiz. This will allow us to record your credit for participation anonymously.

This content is neither created nor endorsed by Google.

Google Forms
