# Supplementary material for: Creative, Socially-Minded Communication Project on Neurodivergences Increases Self-Reported Content Knowledge, Collaboration Skills, and Personal Allyship
Source: J Undergrad Neurosci Educ. 2026 Jun 4;24(2):71–9. doi: 10.59390/001c.162900 (PMC13625903; doi:10.59390/001c.162900)
Supplement: Supplementary file 3 [file junejournal_2026_24_2_162900_347745.docx]

**Supplementary material 2**

**A priori framework analysis tags:** Each set of potential themes/topics was extracted from a framing document. This is not an exhaustive list.

*Tags from Peer Feedback Document*

- Organization
  - Structure:function association in design
- Balancing scientific and social concepts
- Audience
  - Appropriateness
  - Efficacy
  - Engagement
  - Relatability
- Integration of scientific information
- Appreciation of neurodiversity
- Using examples, metaphors, and analogies
- Content knowledge of neurodivergent brain
- Sense of importance of and interest in neurodiversity research
- Knowledge of researchers
- Infographics, graphical representations
- Identifying meaningful vs. filler information
- Supporting scientific claims
- Clarity of statements
- Use of individual strengths
- Constructive feedback
- Inclusivity
- Respectful language

*Tags from Project Description Document*

- general knowledge of neurodiversity
- Characteristics of neurodivergence
- Current understanding within the scientific community
- Use of individual strengths
- Supportive feedback process
- Collaboration within and between groups
- Simplifying complex topics
- Audience
  - Age-appropriateness
  - Relatability
- Integration of visual and creative elements
- Understanding of societal relevance
- Developing advocacy understanding
- Development of ally identity
- Comfort with open-ended tasks

**Final tag array:** initial tags were merged into these overarching categories; additional tags were added as data was reviewed & analyzed

- Structure/Function in Design

- Balancing Science/Social

- Audience Appropriateness

- Knowledge Depth

- Knowledge Breadth

- Content Knowledge

- Interest in Neurodiversity

- Supporting Claims

- Clarity

- Individual Strengths

- Collaboration

- Creativity

- Advocacy

- Open-ended tasks

- Empathy

- Difference

- Team Expectations

- Communication

- Research Skills

- Pathology framework

- Career

- Ethics

- Positive Attitudes Towards Neurodiversity

- Societal Barriers

- Summarizing
